# Effect of Lactulose on the Intestinal Flora of Elderly Constipation Patients With Chronic Renal Insufficiency

**DOI:** 10.1002/agm2.70086

**Published:** 2026-06-15

**Authors:** Jianxia Ma, Jun Wang, Tianran Han, Zhifang Shao, Weiqi Zhang, Ke Wang, Qinlian Zeng, Kangwei Liu, Yuanwen Chen, Songbai Zhen, Jianfeng Yao

**Affiliations:** ^1^ Department of Gastroenterology Hua Dong Hospital of Fu Dan University Shanghai China; ^2^ Shanghai Gonghui Hospital Shanghai China; ^3^ Shanghai Ren Shou Tang Wenjin Nursing Home Shanghai China

**Keywords:** chronic constipation, elderly, gut microbiota, lactulose, renal insufficiency

## Abstract

**Objectives:**

To evaluate the efficacy of lactulose in the treatment of elderly constipation patients with chronic renal insufficiency and its influence on renal function.

**Methods:**

They were divided into the lactulose group (*n* = 30) and polyethyleneglycol (PEG) group (*n* = 26). The lactulose group was given 15 mL oral lactulose qd × 4 weeks, and the PEG group was given PEG 4000 powder 10 g qd × 4 weeks. Wexner constipation score and Bristol stool type were recorded before and after the intervention. To evaluate the efficacy of lactulose and PEG in improving constipation in elderly patients with chronic constipation with renal insufficiency.

**Results:**

Both lactulose and PEG could improve the symptoms of constipation, and no significant difference was observed (*p* = 0.995). After lactulose treatment for 4 weeks, the serum levels of creatinine, uric acid, and IL‐6 reduced significantly (*p* < 0.050), but they remained unchanged after treatment with PEG for 4 weeks (*p* > 0.050). At the genus level, the abundance of *Anaerofustis stercorihominis* (related to the production of short‐chain fatty acids) increased markedly (*p* < 0.050), and the abundance of Halomonadaceae (related to the production of uremic toxins), Eikenella (one of the opportunistic pathogenic bacteria), and Sphingomonas (related to nitrogen metabolism) reduced significantly after lactulose treatment.

**Conclusion:**

Lactulose can improve renal function, mild inflammation, and gut microbiota in these patients. We speculate that the improvement of renal function after lactulose treatment may be related to the improvement of gut microbiota and systemic mild inflammation.

## Graphical abstract

This study investigates the regulatory effects of lactulose and polyethylene glycol (PEG) on the composition of gut microbiota in elderly constipation patients with chronic renal insufficiency by comparing the changes in microbial abundance. Both lactulose and PEG significantly modulate the gut microbiota structure, though their specific mechanisms and trends of microbial shifts differ. We speculate that the improvement of renal function after lactulose treatment may be related to the improvement of gut microbiota and systemic mild inflammation.

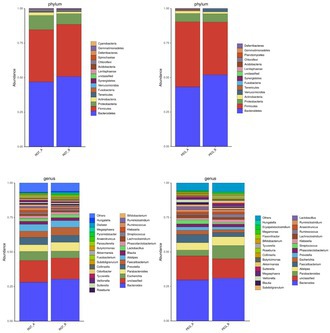

## 1 Introduction

Chronic constipation in the elderly is very common in clinical practice, and its incidence increases with age. In China, it has been reported that the incidence of chronic constipation is 23.0% in people over 70 years old [1]. Constipation is one of the common gastrointestinal symptoms in patients with chronic renal insufficiency.

More stringent dietary restrictions, fluid intake restrictions, accumulation of uremic toxins in the body, and changes in gut microbiota, all of which are commonly present in patients with chronic renal insufficiency, may further increase the prevalence of constipation in this particular population [2]. Although it is generally recognized that the prevalence of constipation in patients with renal insufficiency is higher than that in the general population, it is only in recent years that studies have begun to investigate whether the presence of constipation worsens kidney function and whether the improvement of constipation improves kidney function. The mechanism has not been elucidated. Therefore, this study evaluated the efficacy of lactulose on constipation symptoms in elderly patients with chronic constipation combined with renal insufficiency and its influence on renal function and explored the possible mechanism of lactulose in the treatment of patients with constipation combined with renal insufficiency from the perspective of intestinal microecology, providing a new method to promote the basic treatment of constipation patients. The potential mechanism underlying the protective effect of lactulose on constipation, and renal function of patients with CRI was explored from the perspective of intestinal microecology, which may provide new strategies for the treatment of constipation.

## 2 Subjects and Methods

### 2.1 Subjects

Outpatients and inpatients were recruited from HuaDong Hospital of Fudan University, Shanghai Renshoutang Wenjin Nursing Home, Shanghai Jinfu Nursing Home, and Health Service Center of Changshou Street between December 2021 and October 2022. A total of 60 patients (4 cases lost to follow‐up) were randomly divided into the lactulose group (*n* = 30) or the PEG group (*n* = 26). All enrolled subjects signed informed consent, and the study was approved by the ethics committee of Huadong Hospital (2020K001).

### 2.2 Inclusion Criteria

Patients were ≥ 60 years old; constipation was diagnosed according to the Rome IV diagnostic criteria for chronic functional constipation; patients met the diagnostic criteria for chronic renal insufficiency, and the estimated glomerular filtration rate (eGFR) was 30–60 mL/min for more than 3 months.

### 2.3 Exclusion Criteria

Patients had serious primary diseases of cardiovascular, cerebrovascular, digestive, urinary, and hematopoietic systems; patients were allergic to multiple drugs. Patients had allergic diseases or were allergic to multiple drugs; Patients received treatment of renal diseases with drugs (such as opioid analgesics, tricyclic antidepressants, anticholinergics, and others) that may cause constipation within the prior month; patients were treated with probiotics, prebiotics, synbiotics, antibiotics or received fecal bacteria transplantation in the prior month.

### 2.4 Treatments

In the lactulose group, patients were orally treated with lactulose (Beijing Hanmei Pharmaceutical Co. Ltd.; H20065730; 100 mL) once daily (15 mL) for 4 weeks. In the polyethylene glycol (PEG) group, PEG (Hunan Warner Pharmaceutical Co. Ltd.; H20052078; 10 g) was administered once daily (10 g) for 4 weeks. General information: age, gender, height, weight, BMI, and other data were recorded.

### 2.5 Symptoms of Constipation

The Wexner score and Bristol score were recorded before intervention and 4 weeks after drug intervention (Table 1). The stool form was divided into hard stool form (Bristol 1–2), normal stool form (Bristol 3–5), and loose stool form (Bristol 6–7).

**TABLE 1:** Bristol stool form scale.

Type 1	Separate hard lumps, like nuts
Type 2	Sausage‐shaped but lumpy
Type 3	Like a sausage or snake but with cracks on its surface
Type 4	Like a sausage or snake, smooth and soft
Type 5	Soft blobs with clear‐cut edges
Type 6	Fluffy pieces with ragged edges, a mushy stool
Type 7	Watery, no solid pieces

### 2.6 Laboratory Examinations

The serum levels of creatinine (CR), uric acid (UA), hypersensitive C‐reactive protein (hs‐CRP), β2 microglobulin (β2‐MG), and interleukin‐6 (IL‐6) were detected. The eGFR was calculated according to the Chronic Kidney Disease Epidemiology Collaboration formula (CKD‐EPI).

### 2.7 Microbiota Detection

The stool samples (5 g) were collected into an AxyPrepDNA gel recovery kit 1 day before and after treatment and stored at −80°C within 2 h. All the fecal samples were processed for the establishment of the gene library as follows: (1) genomic DNA extraction; (2) PCR amplification; (3) AxyPrepDNA gel recovery kit; (4) real‐time fluorescence quantification using the FTC‐3000TM real‐time PCR instrument. After the establishment of the gene library, 16S rRNA high‐throughput sequencing and bioinformatics data analysis were performed.

### 2.8 Assessment of Therapeutic Efficacy

The Wexner score was used to evaluate the efficacy of the treatment of constipation. Efficacy index = (Wexner score before treatment minus Wexner score after treatment)/Wexner score before treatment × 100%. The therapeutic efficacy was divided into four types: (1) ineffectiveness: efficacy index < 30%; (2) effective: 30% ≤ efficacy index < 70%; (3) obvious effect: 70% ≤ efficacy index < 95%; (4) recovery: efficacy index ≥ 95%. Effectiveness rate = (number of patients with 2, 3, 4)/total number of patients × 100% [3].

### 2.9 Statistical Analysis

The R Foundation for Statistical Computing (Vienna, Austria) and SPSS26.0 (SPSS, Chicago, IL, USA) were used for the statistical analysis. The Shapiro–Wilk test was applied to the experimental data.

If both groups were subject to a normal distribution, the mean (standard deviation) was used for statistical description; the independent sample *t*‐test was used for comparison between groups, and the paired sample *t*‐test was used. Otherwise, the median (lower quartile and upper quartile) was used for statistical description. The independent sample Wilcoxon rank sum test was used for comparison between groups, and the paired Wilcoxon rank sum test was used for comparison before and after the same group. The number of cases (percentage) was used for the statistical description of the classified data, and the *χ* ^2^ test was used for comparison. *p* < 0.05 was considered statistically significant. GraphPad Prism 8.0.2 and the R language 3.6.0 were used for drawing.

## 3 Results

### 3.1 General Characteristics

A total of 30 patients were included in the lactulose group, including 18 females and 12 males. The mean age was (87.03 ± 6.61) years, the mean height was (162.67 ± 8.13) cm, the mean weight was (64.67 ± 9.18) kg, and the mean BMI was (24.58 ± 4.09) kg/m^2^.

A total of 26 people were included in the PEG group, including 17 females and 9 males. The mean age was (87.77 ± 5.91) years, the mean height was (162.31 ± 8.56) cm, the mean weight was (63.12 ± 6.31) kg, and the mean BMI was (24.04 ± 2.54) kg/m^2^. There were no significant differences in age, sex, height, weight, and BMI between the two groups (*p* > 0.050), indicating that the lactulose group and the PEG group were comparable in general demographic characteristics.

### 3.2 Wexner Score Before and After Treatment

The Wexner score was used to assess the efficacy of lactulose and PEG in the treatment of chronic constipation in elderly patients with renal insufficiency. In the lactulose group, effectiveness was noted in 1 patient and remission in 22 patients with an overall effectiveness rate of 76.67%. In the PEG group, effectiveness was found in 1 patient and remission in 19 patients with an overall effectiveness rate of 76.92%. There was no significant difference in the overall effectiveness rate between the two groups (*p* = 0.995).

### 3.3 Bristol Stool Form Before and After Treatment

In the lactulose group, hard stool form was noted in 18 patients (60%), normal form in 8 (26.67%), and mushy form in 4 (13.33%). After lactulose treatment, hard stool form was found in 2 patients (6.67%), normal form in 22 (74.33%), and mushy form in 6 (20%). The proportion of hard stools decreased from 60% to 6.67%, the proportion of normal stools increased from 26.67% to 74.33%, and the proportion of loose stools increased from 4% to 20% (*p* < 0.001).

In the PEG group, the hard stool form was noted in 19 patients (73.08%), normal form in 6 (23.08%), and loose form in 1 (3.85%). After PEG treatment for 4 weeks, the hard stool form was found in 4 patients (15.38%), the normal form in 20 (76.92%), and the mushy form in 2 (7.69%). The proportion of hard stool decreased from 73.08% to 15.38%, the proportion of normal stool increased from 23.08% to 76.92%, and the proportion of loose stool increased from 3.85% to 7.69% (*p* < 0.001).

These findings indicate that both lactulose and PEG could improve the stool characteristics of elderly chronic constipation patients with renal insufficiency: the stool form was improved, and stool evacuation became smooth.

### 3.4 Laboratory Examinations Before Treatment

There were no significant differences in routine blood tests, inflammation index, and kidney function between the two groups (*p* > 0.050). The median serum hs‐CRP level was > 3 mg/L, and the median value of IL‐6 level was > 7 pg/mL in both groups before the intervention, indicating mild inflammation in the elderly patients with constipation and renal insufficiency (Table 2).

**TABLE 2:** Laboratory examinations before treatment in two groups.

Variables	Lactulose group (*n* = 30)	PEG group (*n* = 26)	*p*
BUN (mmol/L)	7.50 (6.15, 10.30)	9.50 (6.93, 11.77)	0.165
Cr (μmol/L)	94.55 (82.45, 119.70)	100.00 (78.00, 114.00)	0.954
UA (μmol/L)	330.33 ± 80.34	334.30 ± 96.65	0.867
β2‐MG (mg/L)	4.06 (2.96, 5.24)	4.45 (2.82, 5.91)	0.681
Cys (mg/L)	1.66 ± 0.51	1.65 ± 0.45	0.923
hs‐CRP (mg/L)	4.43 (1.49, 6.07)	4.17 (2.90, 7.23)	0.293
IL‐6 (Pg/mL)	8.70 (5.18, 14.00)	7.90 (6.38, 15.10)	0.945
ALT (U/L)	13.20 (7.75, 16.68)	10.80 (6.78, 18.65)	0.645
AST (U/L)	17.30 (14.30, 19.68)	16.15 (12.91, 20.2)	0.480
TBIL (μmol/L)	7.30 (5.73, 11.60)	8.90 (6.01, 12.77)	0.755
WBC (10^9^/L)	6.27 (4.97, 8.25)	5.86 (5.24, 7.06)	0.501
N (%)	60.25 (55.39, 65.22)	64.17 (57.75, 68.05)	0.227
L (%)	25.55 (21.74, 30.80)	23.38 (19.43, 27.78)	0.139
RBC (10^12^/L)	4.02 ± 0.65	3.83 ± 0.54	0.240
Hb (g/L)	123.01 ± 18.14	118.00 ± 15.96	0.281
PLT (10^9^/L)	205.07 ± 65.01	202.78 ± 46.45	0.882
FBG (mmol/L)	4.63 (3.75, 5.19)	4.70 (3.80, 5.66)	0.669
TC (mmol/L)	3.98 (3.51, 4.80)	3.99 (3.49, 4.61)	0.974
TG (mmol/L)	1.05 (0.87, 1.97)	1.15 (0.87, 1.38)	0.928

### 3.5 Kidney Function Before and After Treatment

In the lactulose group, after lactulose treatment, the serum CR level decreased significantly (before: 94.55 (82.45–119.70) μmol/L; after: 87.85 (74.10–111.73) μmol/L) (*p* < 0.001); serum UA level significantly decreased (before: 330.33 ± 80.34 vs. after: 292.57 ± 81.12 μmol/L) (*p* = 0.003) (Table 3). In the PEG group, the serum levels of urea nitrogen, CR, and UA remained unchanged after PEG treatment as compared to those before treatment (Table 4).

**TABLE 3:** Kidney function before and after lactulose treatment.

Variables	Before (*n* = 30)	After (*n* = 30)	*p*
BUN (mmol/L)	7.50 (6.15, 10.30)	6.88 (5.19, 10.33)	0.109
Cr (μmol/L)	94.55 (82.45, 119.70)	87.85 (74.10, 111.73)	< 0.001***
UA (μmol/L)	330.33 ± 80.34	292.57 ± 81.12	0.003**
β2‐MG (mg/L)	4.06 (2.96, 5.24)	4.11 (2.69, 5.48)	0.275

** *p* < 0.01.

*** *p* < 0.001.

**TABLE 4:** Kidney function before and after PEG treatment.

	Before (*n* = 26)	After (*n* = 26)	*p*
BUN (mmol/L)	9.40 ± 3.48	9.63 ± 3.78	0.809
Cr (μmol/L)	102.00 ± 27.04	105.98 ± 40.67	0.551
Ua (μmol/L)	334.30 ± 96.65	317.58 ± 114.92	0.464
β2‐MG (mg/L)	4.45 (2.82, 5.91)	4.38 (3.11, 6.63)	0.121

### 3.6 Composition of Fecal Microbiota

**FIGURE 1:**
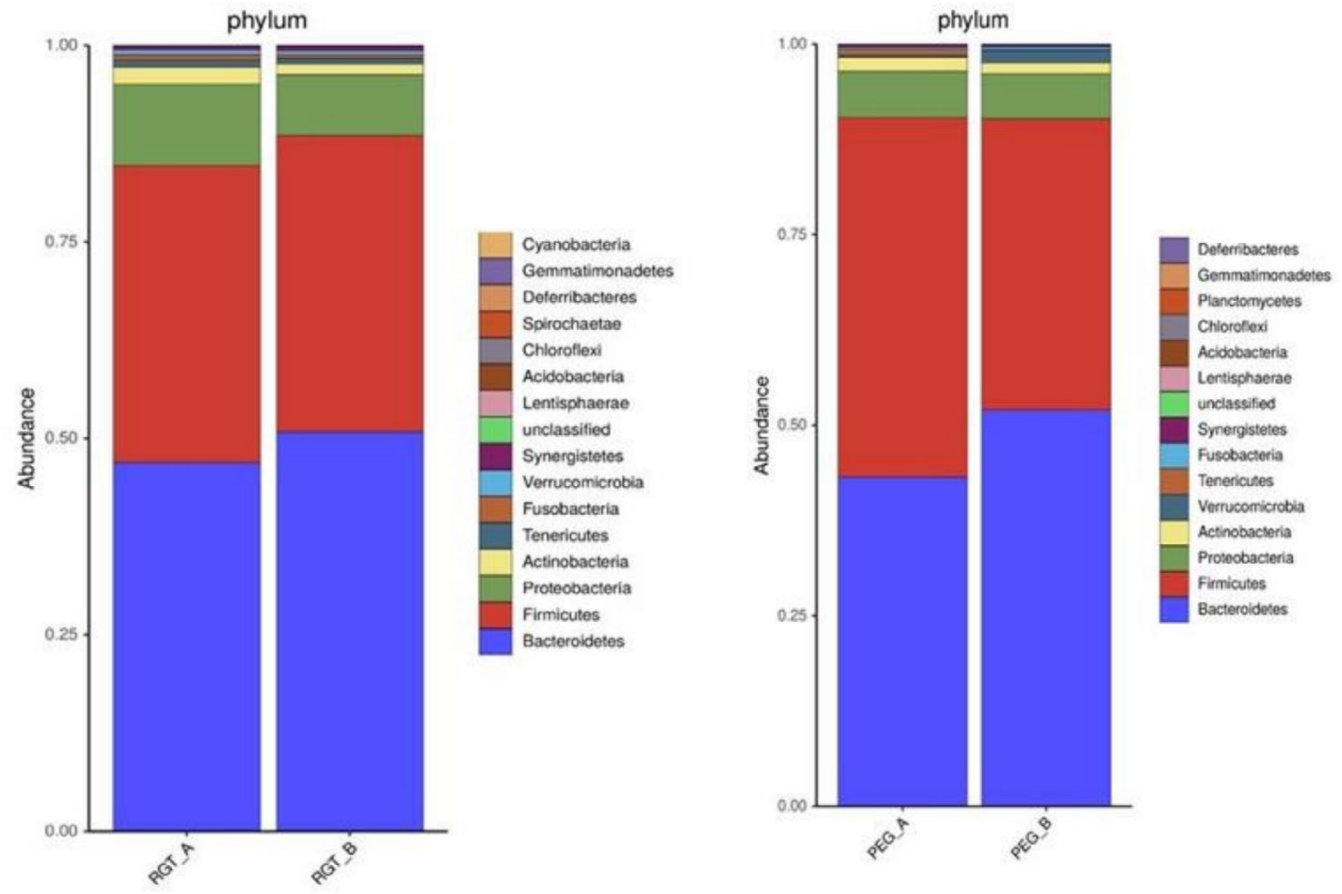
Abundance of fecal microbiota in the lactulose group(Left) and abundance of fecal microbiota in the PEG group (right).

In the phylum level, after lactulose treatment, the abundance of Bacteroidea tended to increase at the phylum level, while the abundance of Proteobacteria and Actinobacteria tended to decrease, although no significant differences were observed (*p* > 0.05) (Figure 1A). In the PEG group, at the phylum level, the abundance of Bacteroidetes showed an increasing trend, while the abundance of Firmicutes showed a decreasing trend, although no significant differences were observed (*p* > 0.05) (Figure 1B).

**FIGURE 2:**
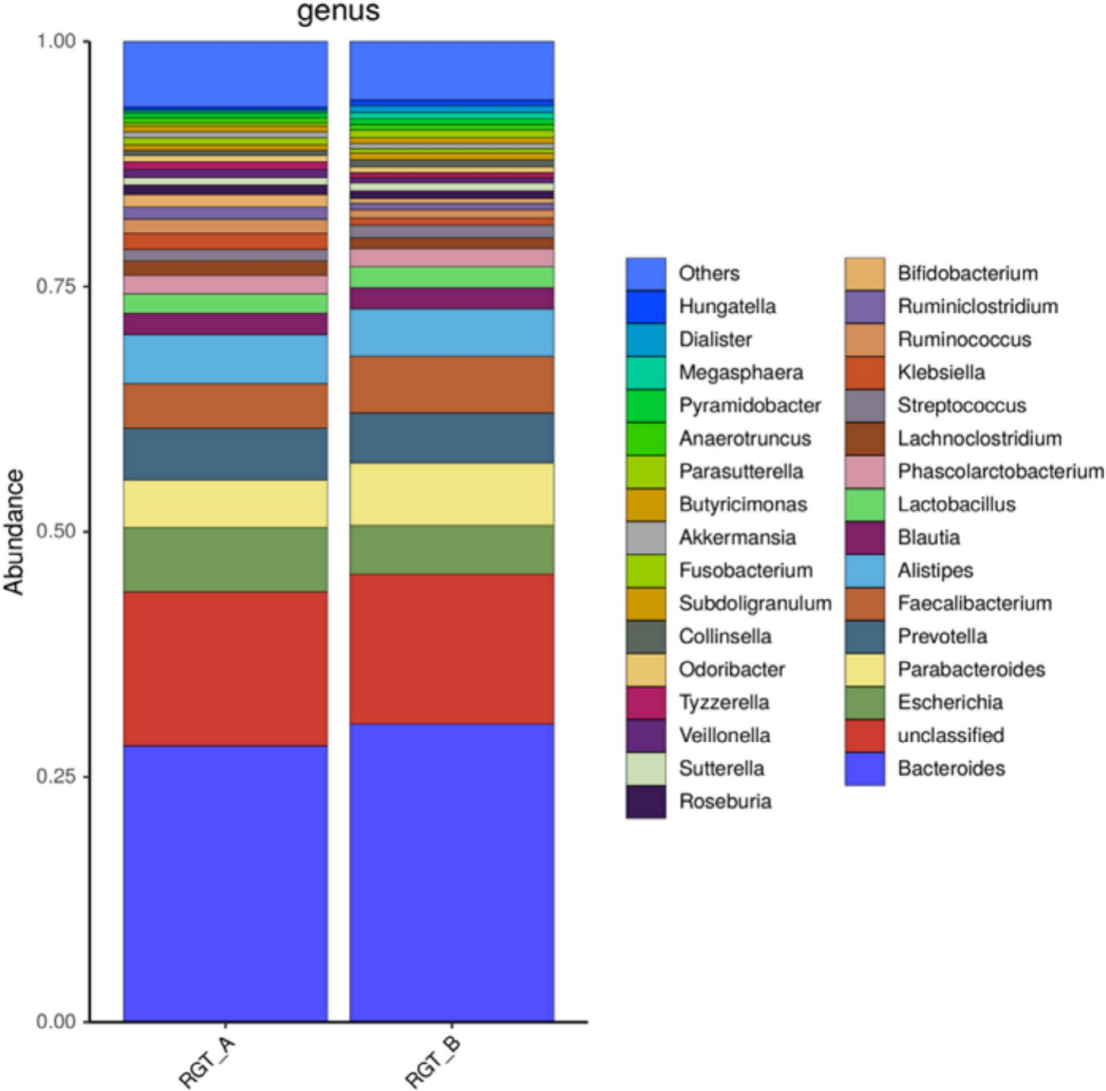
Abundance of fecal microbiota before and after lactose treatment.

**FIGURE 3:**
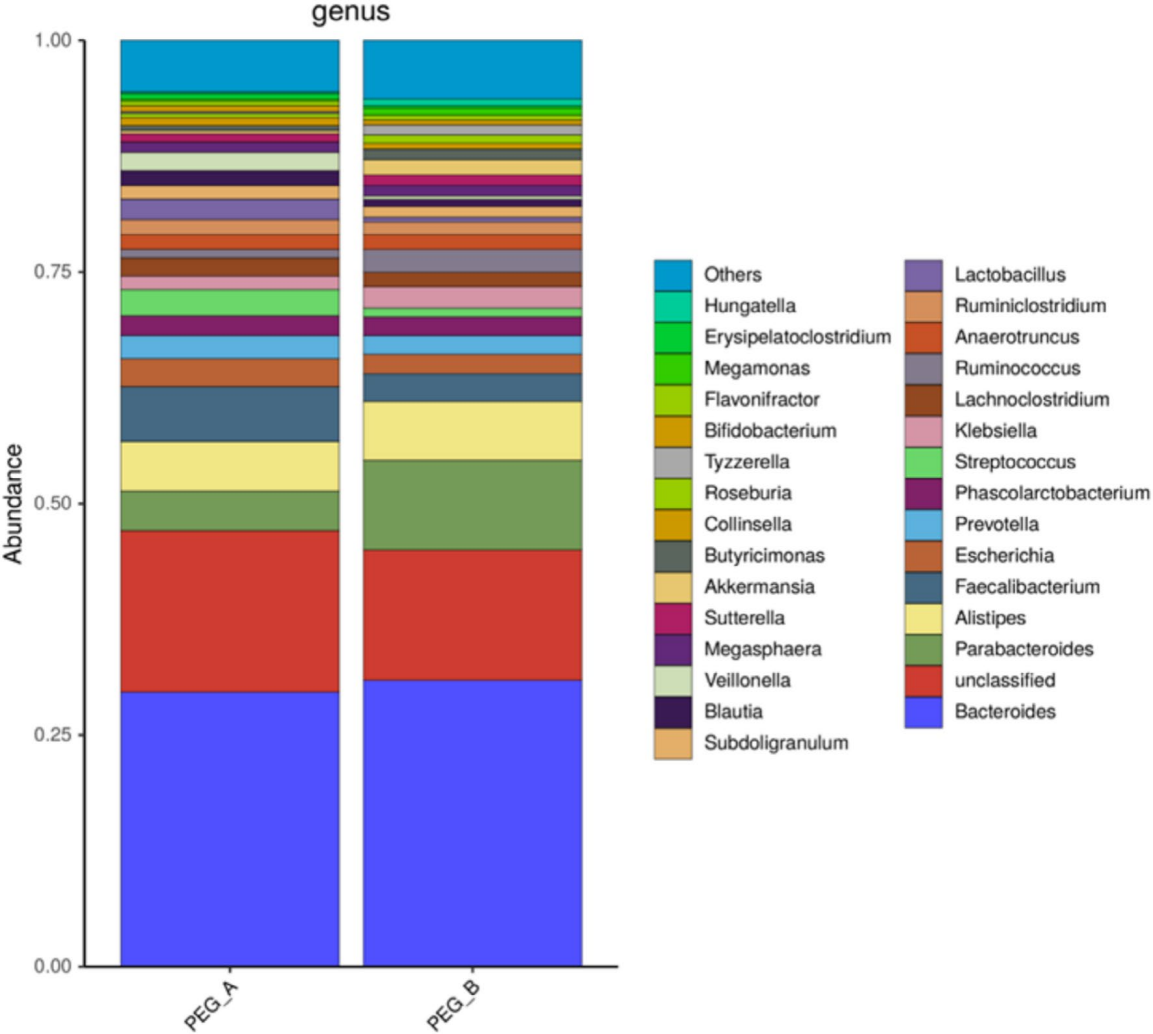
Abundance of fecal microbiota before and after PEG treatment.

In the genus level, the compositions of fecal microbiota before and after lactulose treatment are shown in Figure 3. Among them, Bacteroides, unclassified genera, and Escherichia accounted for about 50% of the total microbiota; the remaining 29 genera with relatively high abundance accounted for about 45%; and other genera accounted for about 5%. As shown in Figure 2, the abundance of Bacteroidei showed an increasing trend, while the abundance of Escherichia tended to decrease, although no significant difference was observed (*p* > 0.05).

After treatment, the abundance of Anaerofustis (related to the production of short‐chain fatty acids [4]) increased significantly, but the abundance of Halomonas (related to the production of uremic toxins), Paeniglutamicibacter, Eikenella (opportunistic pathogenic bacteria), and Sphingomonas (related to nitrogen metabolism) reduced significantly.

The compositions of fecal microbiota at the genus level before and after PEG treatment are shown in Figure 3. Bacteroides, unclassified genera, and Escherichia accounted for about 52% of total microbiota; the remaining 29 genera with relatively high abundance accounted for about 43%; and other genera accounted for about 5%. In addition, the abundance of Parabacteroides (increase with the progressive deterioration of kidney function [5]), Robinsoniella (new pathogenic bacteria), Catabacter, Actinospica, and Amycolatopsi increased significantly, but the abundance of Proteus reduced markedly (*p* < 0.050).

## 4 Discussion

Chronic constipation and chronic kidney disease are the most common groups in the elderly. Currently, little is known about the efficacy of laxatives on constipation in elderly patients with renal insufficiency.

Lactulose is a synthesized disaccharide and a common osmotic laxative in clinical practice. In the colon, it causes an osmotic gradient and, under osmotic pressure, promotes the softening of the stool, making it easier to move through the intestine. Lactulose can also increase the amount of intestinal probiotics such as Lactobacillus and Bifidobacterium. The lactulose may be metabolized by bacteria in the colon into acetic acid and lactic acid, increasing the colon acidity and promoting peristalsis, which is ultimately helpful for fecal evacuation [6]. PEG 4000 is also a commonly used osmotic laxative. PEG can increase the water content of feces, thereby softening the stools and making them easier to evacuate.

Lactulose can also increase the number of intestinal probiotics such as Lactobacillus and Bifidobacterium, and through the action of bacteria in the colon, lactulose can be converted into acetic acid and lactic acid, resulting in increased colon acidity and promotion of peristalsis, which ultimately contributes to fecal excretion [6]. PEG 4000 powder is also a commonly used osmotic laxative, which is not absorbed in the gut and is not broken down by gut bacteria. PEG can increase the water content of stool, which softens stool and makes it easier to excrete.

In the present study, the subjects were divided into a lactulose treatment group and a PEG treatment group, and the effect on constipation was assessed based on the symptoms of constipation (Wexner score) and stool form in elderly patients. Our results showed the Wexner score and stool form were improved significantly in these patients after treatment with lactulose or PEG. In our study, hCRP and IL‐6 in elderly patients with chronic constipation and renal insufficiency were higher than normal levels, indicating mild inflammation in these patients.

In the present study, lactulose treatment for 4 weeks significantly reduced serum levels of CR, UA, and IL‐6 (*p* < 0.050), but kidney function and inflammatory parameters remained unchanged after PEG treatment in these patients.

In patients with kidney disease, some factors (such as colonic congestion and edema, slow colonic movement, use of antibiotics, and reduced dietary fiber intake) may affect the tight connection between intestinal epithelial cells of the colon, leading to increased intestinal permeability and causing translocation of bacterial metabolites through the intestinal barrier, which results in systemic inflammation and immune response, further deteriorating kidney function [7]. In our study, the serum levels of Cr and IL‐6 significantly decreased in elderly patients with chronic constipation and renal insufficiency after lactulose treatment. In the following experiment, their gut microbiota was investigated, aiming to explore the potential mechanism underlying the therapeutic effect of lactulose.

We indicated that, after lactulose treatment, the intestinal flora changed. To date, among studies investigating the intestinal microbiota after treatment targeting the intestinal microbiota, none have reported significant changes in the alpha or beta diversity in elderly patients after probiotic, prebiotic, or synbiotic treatment [8], which is consistent with our finding. Although the alpha or beta diversity of gut microbiota was not altered in the present study, our results showed that the abundance of some microbiota changed significantly after lactulose treatment.

In patients with renal insufficiency, intestinal microbiota imbalance is mainly manifested by an increase in protein fermentation and uremic toxins in the intestine, as well as a decrease in carbohydrate fermentation and beneficial metabolites (such as SCFAs). The progressive deterioration of kidney function can lead to the secretion of urea into the intestine, which may cause the excessive growth of urease‐containing bacteria. The excessive production of uremic toxins increased and damaged the intestinal mucosal barrier function [9]. Vaziri et al. [10] indicated that, as compared to healthy controls, the abundance of pathogenic bacteria (such as actinomycetes, Firmicutes, and Proteobacteria) in the intestine of patients with renal insufficiency significantly increased. Our results showed that after lactulose treatment, the abundance of Firmicutes (37.73% vs. 37.71%), Proteobacteria (10.37% vs. 7.71%), and Actinobacteria (2.16% vs. 1.39%) tended to reduce, although no significant difference was observed.

Our study showed that, after lactulose treatment, the abundance of halomonas (related to the production of uremic toxins), sphingomonas (related to nitrogen metabolism), and Eikenella (opportunistic pathogenic bacteria) significantly decreased (*p* < 0.05). However, in the PEG group, the abundance of pathogenic bacteria or urease‐producing bacteria was not found to be reduced. Instead, the abundance of Porphyromonaceae (related to the increased intestinal permeability [11]), Parabacteroides (increased with the progressive deterioration of kidney function [5]), and some opportunistic pathogenic bacteria significantly increased (*p* < 0.05). The effect of PEG as a laxative on the gut microbiota is only used for intestinal preparation, and it is believed that PEG has little influence on the gut microbiota.

Some studies also indicate [12] that lactulose treatment did not significantly increase the abundance of probiotics (such as Lactobacillus and Bifidobacterium) in patients with chronic constipation. Our study also failed to identify an increase in probiotics after lactulose treatment in patients with chronic constipation and renal insufficiency. This may be explained as follows: (1) Generally, the amount of Bifidobacteria and Lactobacilli reduces significantly [13], which was also observed in patients with renal insufficiency in our study. (2) The pathways related to carbohydrate metabolism and amino acid synthesis are reduced in the elderly population, and the abundance of *Enterococcus faecalis*, Bacteroides, and SCFAs‐producing microbiota in the elderly patients is significantly lower than in the young population [9]. (3) There is a significant individual difference in the gut microbiota [14], and the small sample size limits the performance of testing for the difference in the abundance of these probiotics.

Although significant change was not observed in typical probiotics, the abundance of *Anaerofustis stercorihominis* (related to the production of short‐chain fatty acids) increased significantly in these patients (*p* < 0.050) [14], which was not observed in the PEG group. Therefore, we speculate that lactulose may reduce the abundance of harmful gut bacteria to improve the intestinal microbiota.

In future studies, studies with large sample sizes, more interventions, and long follow‐up are needed to confirm our findings, and the changes in enterogenous uremic toxins and intestinal mucosal barrier function will be more precisely investigated, aiming to provide a theoretical basis for the clinical treatment of chronic constipation in elderly patients with renal insufficiency.

## Author Contributions

J.M. and J.W. designed the study. T.H., Z.S., and W.Z. participated in data collection, collation, and analysis. K.W., Q.Z., and K.L. were in charge of supervising the entire research process. J.M. and Y.C. conducted manuscript writing. S.Z. and J.Y. provided comments on the previous versions of the manuscript and read and approved the final version.

## Funding

This study was funded by Huadong Hospital Key Specialty Inflammatory Bowel Disease Project (2021, ZDZB2221), Huadong Hospital Key Discipline Construction of Gastroenterology (2022, ZDXK2213), and National Natural Science Foundation of China (82270620).

## Ethics Statement

All enrolled subjects signed informed consent, and the study was approved by the ethics committee of Huadong Hospital (2020K001).

## Conflicts of Interest

The authors declare no conflicts of interest.

## Data Availability

The datasets used and/or analyzed during the current study are available from the corresponding author on reasonable request.
